# Preservation of urethra devoid of corpus spongiosum in patients undergoing urethroplasty

**DOI:** 10.4103/0970-1591.32083

**Published:** 2007

**Authors:** Amilal Bhat

**Affiliations:** Department of Urology, S. P. Medical College, Bikaner, India

Dear Sir,

I read with interest article by Sarin and Manchanda.[1]

The issues which need to be resolved in the preservation of hypoplastic urethra:

1. Correction of chordee
  I think authors had been lucky to correct the chordee by simple penile degloving. In my experience such patients of mid-penile and proximal penile hypospadias with hypoplastic urethra of 1.92 cm have significant chordee which is unlikely to be corrected by penile degloving. The authors have not mentioned whether they performed Gitte's test or not which is a sure way to confirm correction of chordee. Mobilization of hypoplastic and proximal urethra along with corpus spongiosum is my preferred choice for correction of chordee in such cases or alternatively plication procedures can be performed. The added advantage with mobilization of urethra and corpus spongiosum is easy and tension-free tubularization of urethral plate.
2. Rational approach in utilization of hypoplastic urethra
  This thin distal urethra is devoid of corpus spongiosum as spongiosum is spread lateral in “Y” manner and is attached to the glans.[2] Hypoplastic urethra is adherent to the skin proximal to the meatus, just as in chordee without hypospadias type I of Devine Horton[3] or may be few millimeters bare near the hypospadiac meatus. Resecting more than 1cm urethra may change the location of hypospadiac meatus, distal penile to mid-penile, mid-penile to proximal penile and so on.[4] So preserving the hypoplastic urethra enlarges the scope of the urethral preservation procedures like TIP where the results of urethroplasty are better than replacement urethroplasty which the authors have done rightly. Care is taken during mobilization of the adherent skin as damage to the hypoplastic urethra may lead to urethral fistula.[5] Authors have not highlighted the technique of mobilization of adherent skin from the hypoplastic urethra. Skin mobilization can be facilitated by saline injection at the site of hypoplastic urethra to create the plane of dissection.[6] I use the adrenaline solution (1: 100,000), the same is also used at the site of skin incision.

Preservation of hypoplastic urethra depends on the type of hypospadias, degree of chordee and type of repair chosen. The thin urethra can easily be preserved and utilized in urethral plate preservation procedures like TIP in distal hypospadias with minimal or no chordee. Spongioplasty in these cases will reconstruct the near normal urethra and will help in correction of chordee.[2] It is recommended not to leave any space between the hypoplastic urethra and urethral plate during tubularization to prevent fistula. Conventionally, hypoplastic urethra is resected in proximal hypospadias with chordee or middle hypospadias with severe chordee where chordee correction is unlikely by plication procedures. This urethra is considered less vascular and will increase the chances of anastomotic fistula.[4] The extent of resection of hypoplastic urethra is up to healthy urethra covered with corpus spongiosum and anastomosis should be with healthy well vascularized urethra. To utilize hypoplastic urethra in these cases, I correct chordee by mobilization of urethra and corpus spongiosum and perform tubularized incise plate urethroplasty.

The authors have come across one case with chordee without hypospadias but which type, has not been mentioned. But by the description it looks like type III, where corpus spongiosum is well developed, but only dartos fascia is deficient and chordee correction is possible by penile degloving. Though some authors have advised reconstruction of the entire dysplastic urethra when the paper-thin urethra of type I chordee without hypospadias is associated with an abnormal meatus.[5] But with better understanding of the problems of type I chordee without hypospadias, dissection of hypoplastic urethra along with corpus spongiosum and technique of hydro-dissection,[6] it is possible to mobilize the hypoplastic urethra without damage. If mobilization of hypoplastic urethra and corpus spongiosum with or without dorsal plication corrects the chordee, then spongioplasty over hypoplastic urethra will be a better option than replacement urethroplasty. In case of persistence of chordee or damage to hypoplastic urethra, resection of hypoplastic urethra and replacement of urethroplasty is advised.
